# Recurrent Dystonia Associated With Alcohol Withdrawal in a Young Adult: A Case Report

**DOI:** 10.7759/cureus.117666

**Published:** 2026-10-09

**Authors:** Shreya K Srinivas, Rashmi H Rajur

**Affiliations:** 1 Neurology, Rajarajeswari Medical College and Hospital, Bangalore, IND

**Keywords:** acute dystonia, alcohol cessation, alcohol withdrawal phenomenon, alcohol withdrawal syndrome, dystonia, paroxysmal dystonia

## Abstract

Withdrawal symptoms are common in individuals with chronic alcohol use and may range from autonomic and neuropsychiatric manifestations to seizures and movement disorders, such as tremor and dyskinesia. Dystonia is a rarely reported manifestation of alcohol withdrawal. We report the case of a 35-year-old man with chronic alcohol use disorder (AUD) who developed recurrent, stereotyped dystonic attacks approximately 24 hours after cessation of alcohol intake, with no recurrence during sustained abstinence. This case highlights the differential diagnosis of dystonic episodes occurring in temporal association with the alcohol withdrawal state and discusses the possibility of an alcohol withdrawal-associated phenomenon.

## Introduction

Dystonia in a young adult warrants evaluation for underlying causes, including drug-induced, metabolic, structural, autoimmune, infectious, and genetic etiologies. Alcohol withdrawal is a common clinical syndrome in individuals with chronic alcohol use and typically presents with autonomic hyperactivity, tremor, agitation, anxiety, hallucinations, delirium, or seizures [1]. Although tremor is the most frequent movement disorder associated with alcohol withdrawal, other hyperkinetic disorders, including chorea and dyskinesia, have also been reported. However, when a withdrawal phenomenon presents as dystonia, it warrants evaluation, as it is an exceptionally rare manifestation [2,3].

## Case presentation

A 35-year-old man presented with a four-year history of recurrent episodes of abnormal twisting movements of the body. He reported approximately two to three episodes per month. The episodes typically began with sustained backward twisting of the neck, lateral flexion of the trunk, and flexion at the hips and both knees. These movements were involuntary and episodic, lasting approximately five minutes to two hours, and occurred multiple times during the day. The episodes disappeared during sleep and were not associated with loss of consciousness. The patient’s attacks were stereotyped across all attacks. He had used alcohol for approximately 10 years, predominantly in the form of continuous weekend binge drinking, consuming approximately eight to 10 pints of beer or four to five servings of 60 ml whisky, sometimes both. He reported that his last drink preceded symptom onset by approximately one day. He had not previously experienced severe alcohol withdrawal symptoms except for agitation, nausea, and sweating.

The patient had consulted another physician approximately two years earlier for the same complaint. An interictal electroencephalogram, performed two years earlier, 18 hours after a similar stereotyped movement, was normal (Figure 1); he was then prescribed brivaracetam 50 mg twice daily. There was no resolution of attacks with brivaracetam, and hence, it was subsequently discontinued. There was no history of head injury, significant drug exposure, or other relevant medical or surgical illness. There was no family history of dystonia, tremor, parkinsonism, other movement disorders, or similar episodic abnormal posturing before the onset of symptoms four years earlier.

**Figure 1:**
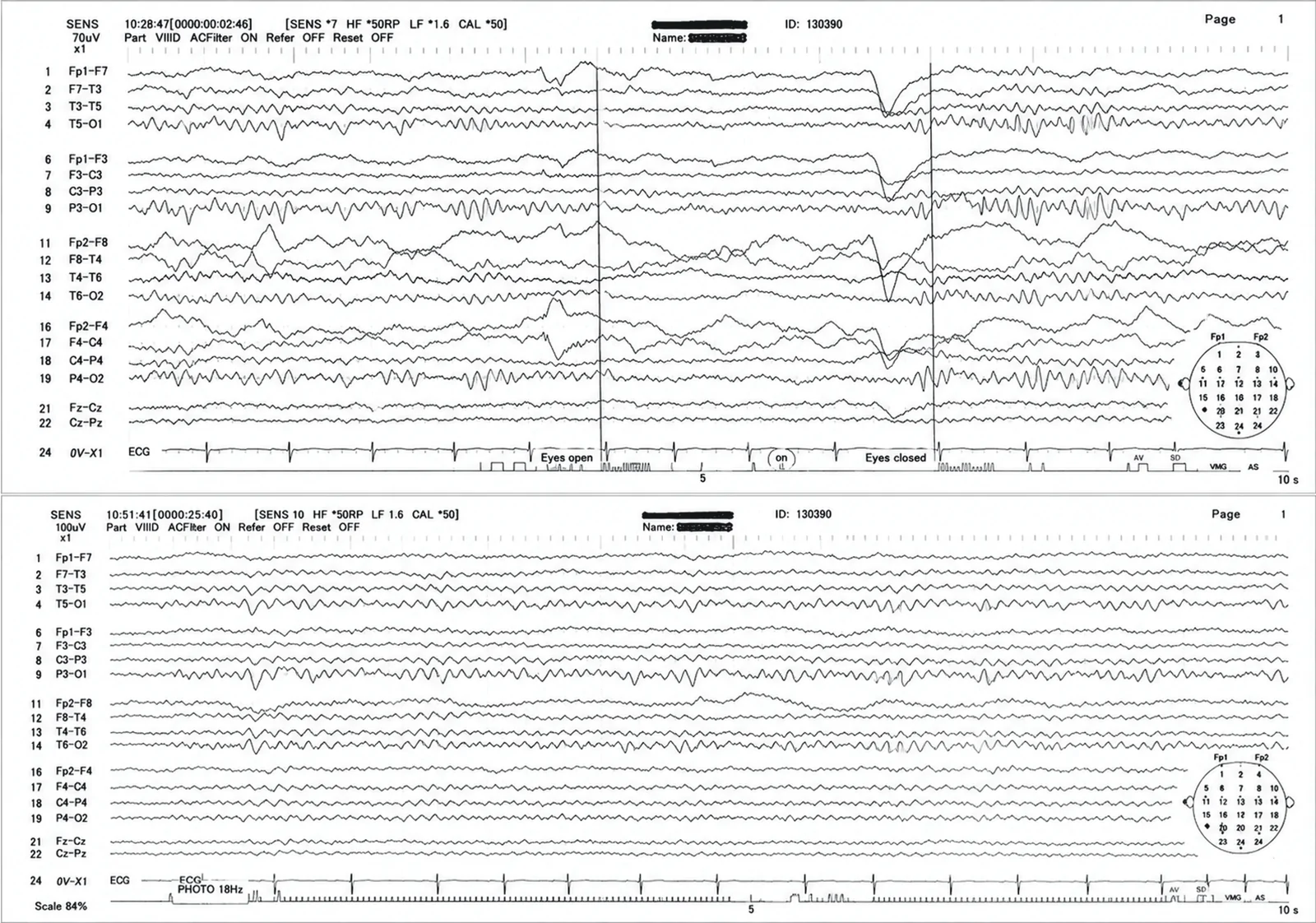
Electroencephalogram performed approximately 18 hours after the dystonic episode EEG was obtained using a bipolar montage. Sensitivity was 7 µV/mm, with a time base (paper speed) of 10 seconds/page. Filters were set at 1.6 Hz (low-frequency/high-pass filter) and 50 Hz (high-frequency/low-pass filter); no notch filter was applied.

On day 1 of the hospital visit, a typical episode was observed. The patient remained conscious and aware, and he exhibited a stereotyped pattern of abnormal posturing, characterized by retrocollis, lateral and forward flexion of the trunk, and flexion at the hip and both knees. This episode lasted approximately 40-50 minutes. There was no loss of consciousness, tongue biting, tonic-clonic movements, urinary or fecal incontinence, or tremor during the episodes. The movements were not rhythmic, not voluntarily suppressible, and did not include repetitive side-to-side neck movements, screaming, dramatic behavioral manifestations, pelvic thrusting, or other prominent hyperkinetic behaviors. He was shifted to the Emergency Department. His pulse rate was 104/min, blood pressure was 130/70 mmHg, respiratory rate was 18/min, and oxygen saturation was 96% on room air. He was afebrile. This attack did not disappear or substantially change with distraction and was neither inducible nor voluntarily modifiable. However, walking or other limb movement exacerbated the posturing, particularly the retrocollis and lateral trunk flexion. The consistent phenomenology across attacks, persistence despite distraction, absence of suppressibility, and absence of other positive functional features were considered clinically relevant and argued against functional dystonia but could not definitively exclude it.

Neurological examination between episodes showed normal higher mental functions and cranial nerves. Motor examination was normal, with preserved deep tendon reflexes except for diminished bilateral ankle jerks. Plantar responses were flexor bilaterally. Sensory examination, gait, and coordination were normal. No dystonia was present between episodes. Magnetic resonance imaging of the brain showed no significant abnormality. Routine laboratory investigations were within normal limits, including hemoglobin 15.5 g/dL, total leukocyte count 6,960/mm³, platelet count 2.45 × 10⁵/mm³, renal function, serum electrolytes, liver function tests, and random blood glucose of 88 mg/dL. Serum ceruloplasmin and 24-hour urinary copper excretion were normal. The laboratory findings are summarized in Table 1. Slit-lamp examination revealed no Kayser-Fleischer rings. EEG monitoring during the current dystonia attack was not performed.

**Table 1:** Laboratory investigations

Investigations	Patient value	Reference range
Hemoglobin	15.5 g/dL	13.0–17.0 g/dL
Total leukocyte count	6,960 /mm³	4,000–11,000 /mm³
Platelet count	2.45 × 10⁵ /mm³	1.50–4.50 × 10⁵ /mm³
Serum urea	22 mg/dL	15–40 mg/dL
Serum creatinine	1.06 mg/dL	0.70–1.20 mg/dL
Serum uric acid	7.1 mg/dL	3.5–7.2 mg/dL
Sodium	140 mmol/L	136–145 mmol/L
Potassium	4.1 mmol/L	3.5–5.1 mmol/L
Calcium	9.2 mg/dL	8.5–10.5 mg/dL
Magnesium	1.7 mg/dL	1.7–2.2 mg/dL
Total bilirubin	0.6 mg/dL	0.2–1.2 mg/dL
Direct bilirubin	0.03 mg/dL	0.00–0.30 mg/dL
AST	22 U/L	10–40 U/L
ALT	19 U/L	7–56 U/L
Alkaline phosphatase	76 U/L	44–147 U/L
Total protein	6.7 g/dL	6.0–8.3 g/dL
Albumin	4.0 g/dL	3.5–5.0 g/dL
Random blood glucose	88 mg/dL	70–140 mg/dL
HbA1c	4.9%	<5.7%
Serum ceruloplasmin	Normal	20–35 mg/dL
24-hour urinary copper	22 µg/24 hours	<50 µg/24 hours

The patient was suspected to have an acute dystonic episode. The patient received a single intravenous dose of pheniramine (2 mL = 45.5 mg), clonazepam 0.5 mg orally at bedtime, intravenous thiamine 100 mg three times daily, IV fluids, and other supportive treatment. The dystonic posturing resolved within approximately two hours, and no further episode was observed during the hospital stay. A psychiatric consultation was obtained during the hospital stay, and the patient was diagnosed with alcohol use disorder (AUD) meeting six of the 11 Diagnostic and Statistical Manual of Mental Disorders, Fifth Edition (DSM-5) criteria for AUD. The CIWA-Ar (Clinical Institute Withdrawal Assessment for Alcohol, Revised) score during the hospital stay was 7, and he was counselled for de-addiction. He was discharged on clonazepam 0.5 mg at night for 15 days and was counselled regarding alcohol cessation. At the two-month follow-up, the patient had resumed alcohol consumption and subsequently developed another similar episode approximately one day after binge alcohol consumption. A video demonstrating the patient's abnormal movements during an episode is shown in the Appendix. He was readmitted and treated with clonazepam 0.5 mg, one tablet at night for five days. At the eight-month follow-up, he had maintained abstinence and remained free of further dystonic episodes.

The recurrence following renewed alcohol exposure and the absence of further episodes during sustained abstinence were considered clinically important observations. Taken together with the observed dystonic posturing and the absence of an identified structural, metabolic, toxic, or drug-induced cause, these findings raised the possibility of an underlying alcohol withdrawal-related mechanism.

## Discussion

Movement disorders associated with alcohol withdrawal are not uncommon, with tremor being the most frequent manifestation. However, other hyperkinetic disorders, including chorea, myoclonus, and dystonia, have also been reported, with alcohol withdrawal-induced dystonia remaining a rare and potentially underrecognized clinical entity [3,4]. The temporal relationship between alcohol cessation and the onset of symptoms, together with the exclusion of structural, metabolic, and drug-induced etiologies, supports a possible association with alcohol withdrawal and the observed movement disorder.

Neiman J et al. [2] established that alcohol withdrawal can rarely produce transient extrapyramidal syndrome, supporting the concept of abrupt alcohol cessation producing basal ganglia dysfunction. The pathophysiology of alcohol withdrawal dystonia remains incompletely understood but is thought to result from neuroadaptive changes following chronic alcohol exposure. Ethanol enhances γ-aminobutyric acid (GABA)-mediated inhibitory neurotransmission while suppressing N-methyl-D-aspartate (NMDA) receptor-mediated excitatory transmission. Chronic alcohol consumption leads to compensatory downregulation of GABA-A receptors and upregulation of NMDA receptors [5]. Abrupt cessation of alcohol may therefore result in a state of neuronal hyperexcitability, potentially contributing to the development of dystonia.

Only a limited number of cases describing dystonia as a manifestation of alcohol withdrawal have been reported. Bijjal et al. described three cases of acute cervical dystonia occurring during alcohol withdrawal, which resolved following treatment with benzodiazepines and anticholinergic therapy, highlighting the reversible nature of this disorder [3]. In a separate report, Prakash and Balhara described dyskinesia as a manifestation of alcohol withdrawal, further supporting the occurrence of hyperkinetic movement disorders in the withdrawal state [4]. Although uncommon, these reports suggest that dystonia may occur as part of the spectrum of alcohol withdrawal-related movement disorders.

The differential diagnosis of recurrent dystonia includes drug-induced dystonia, structural lesions, Wilson’s disease, metabolic abnormalities, autoimmune encephalitis, paroxysmal dyskinesias, and genetic dystonias. Other important considerations in our patient included tonic seizures, functional dystonia, and an underlying idiopathic or genetic dystonia that might be temporarily suppressed by alcohol and become apparent following alcohol cessation. Such alcohol responsiveness has been described in certain inherited movement disorders, including dystonia phenotypes [6]. Paroxysmal dyskinesia was also considered given the episodic nature of the attacks and exacerbation with movement. However, our patient had a 10-year history of AUD, meeting the DSM-5 diagnostic criteria for AUD [7], without any previous history of dystonia or other movement disorder and with a negative family history of an inherited movement disorder. All reported episodes occurred approximately one day after cessation of alcohol intake following binge consumption, while no dystonia was reported or observed during periods of abstinence. Importantly, an identical episode occurred approximately one day after cessation following a subsequent binge episode, two months after the initial attack. This reproducible temporal association with alcohol cessation, together with the absence of a previous history of dystonia and subsequent absence of episodes during sustained abstinence, led us to consider an alcohol withdrawal-related mechanism as a possible etiology. However, psychiatrist assessment and a CIWA-Ar score of 7 documented the presence of withdrawal symptoms during hospitalization but, in isolation, did not establish a causal relationship between alcohol withdrawal and the dystonic episodes.

The possibility of an epileptic etiology was also considered, particularly given the episodic and stereotyped nature of the events. Although the interictal EEG was normal, it was obtained approximately 18 hours after a similar episode and not during a typical attack; therefore, a normal interictal EEG does not exclude an epileptic etiology. The absence of loss of consciousness, tongue biting, incontinence, or other features suggestive of seizures, together with poor response to antiseizure medications, made an epileptic cause less likely, but it could not be definitively excluded. Functional dystonia was another consideration. The attacks were not distractible or suppressible and were not inducible, and no other positive functional signs were identified, which may argue against functional dystonia. However, the absence of these features does not definitively exclude a functional movement disorder. Genetic testing was not performed to evaluate for an underlying inherited or alcohol-responsive movement disorder. As the patient has remained asymptomatic during sustained abstinence, further genetic evaluation was deferred. These limitations preclude definitive exclusion of alternative etiologies and support interpreting the present case as a possible alcohol withdrawal-associated phenomenon.

Benzodiazepines are the mainstay of pharmacological treatment for alcohol withdrawal and are effective in reducing withdrawal symptoms [8]. Recognition of this possible association may help clinicians consider alcohol withdrawal-related movement disorders when evaluating recurrent dystonia in patients with AUD, after other common etiologies have been excluded.

## Conclusions

Acute dystonia is a common clinical presentation. In patients with AUD, a careful evaluation for secondary causes is warranted. After excluding common structural, metabolic, toxic, drug-induced, epileptic, and functional causes, alcohol-responsive movement disorders and a possible alcohol withdrawal-related movement disorder should also be considered in patients with recurrent dystonic episodes temporally related to alcohol cessation.
